# Bone metastatic carcinoma with EGFR amplification and mutation: A case report and literature review

**DOI:** 10.1097/MD.0000000000032615

**Published:** 2023-01-20

**Authors:** Hong-Juan Du, Fang-Fang Chen, Yu Liu, Yu Zhou

**Affiliations:** a Department of Oncology, Chongqing General Hospital, Chongqing, China; b Department of Respiration, Fuling People’s Hospital, Chongqing, China.

**Keywords:** BMCUP, Case report, EGFR, osimertinib

## Abstract

Mutations in the epidermal growth factor receptor (EGFR) gene are highly prevalent in non-small cell lung cancer, while rare in other cancers. Primarily it’s hardly present in bone metastases from cancer of unknown primary (BMCUP). Currently, no specific treatment options for bone metastases from unknown primary cancers exist.

**Patient concerns:**

The right shoulder and back pain of a 72-years-old man had been persistent for 2 weeks and had developed worse on 1 particular day. The right upper arm was compromised, which also hindered the arm’s ability to raise and flex, and nighttime sleep was impacted. After applying the analgesic patch externally, the symptoms did not improve. No coughing or sputum production, chest tightness, shortness of breath, acid reflux, belching, abdominal pain, distension, diarrhea, backache, hematuria, black or bloody feces, or other discomforts appeared over the course of the illness.

**Diagnoses:**

The patient had a particular type of bone metastases from primary cancers with genetic test results indicating EGFR amplification and mutation.

**Interventions:**

A third-generation tyrosine kinase inhibitors drug, oral Osimertinib 80 mg once a day with bisphosphonates anti-bone destruction treatment was performed on schedule.

**Outcomes:**

Following treatment, the patient’s tumor-related symptoms were significantly improved by controlling the disease for up to 11 months and providing great pain relief.

**Lesson:**

EGFR-based genetic testing has emerged as a key measure for targeted therapy in non-small cell lung cancer. However, there are fewer relevant studies for other tumor types like BMCUP. Combined with literature reviews and our report, we provide evidence that targeting EGFR mutations according to the “basket theory” for the treatment of BMCUP is effective.

## 1. Introduction

We report the diagnosis and treatment of a case of bone metastasis from the unknown primary lesion with epidermal growth factor receptor (EGFR) amplification and mutation and combine it with a literature review to discuss it, and to provide reference and help for clinical diagnosis and treatment. The patient has provided written informed consent for the publication of this manuscript and any identifiable images or data.

Cancer of unknown primary (CUP) is a generic term for a group of metastatic tumors that are histologically diagnosed as metastatic but whose primary anatomical site cannot be identified after examination, accounting for approximately 3% to 5% of all new cancers in humans.^[1]^ Bone metastases from cancer of unknown primary (BMCUP) is a type of CUP with a poor overall prognosis, accounting for about 10% of CUP. BMCUP is common in adults, with a median age of 65 to 90 years, and slightly more males than females. Patients with BMCUP usually have a poor prognosis, with a mean survival of 3 to 12 months.^[2]^ Nowadays, the clinical remedy for patients with BMCUP is still controversial, and there are no clear treatment guidelines.

## 2. Consent for publication

The patient provides written informed consent to publish the case.

## 3. Case report

A 72-years-old man presented with a 2-week history of right shoulder and back pain, aggravated for 1 day. The pain was involved in the right upper arm, the function of raising and flexion of the arm were affected, and sleep was affected at night. The symptoms were not relieved after the external application of the analgesic patch. During the course of the disease, there was no cough and sputum, no chest tightness, no shortness of breath and chest tightness, no acid reflux and belching, no abdominal pain, distension and diarrhea, no backache and hematuria, no black stools, no bloody stools, and other discomforts.

**Figure 1.**
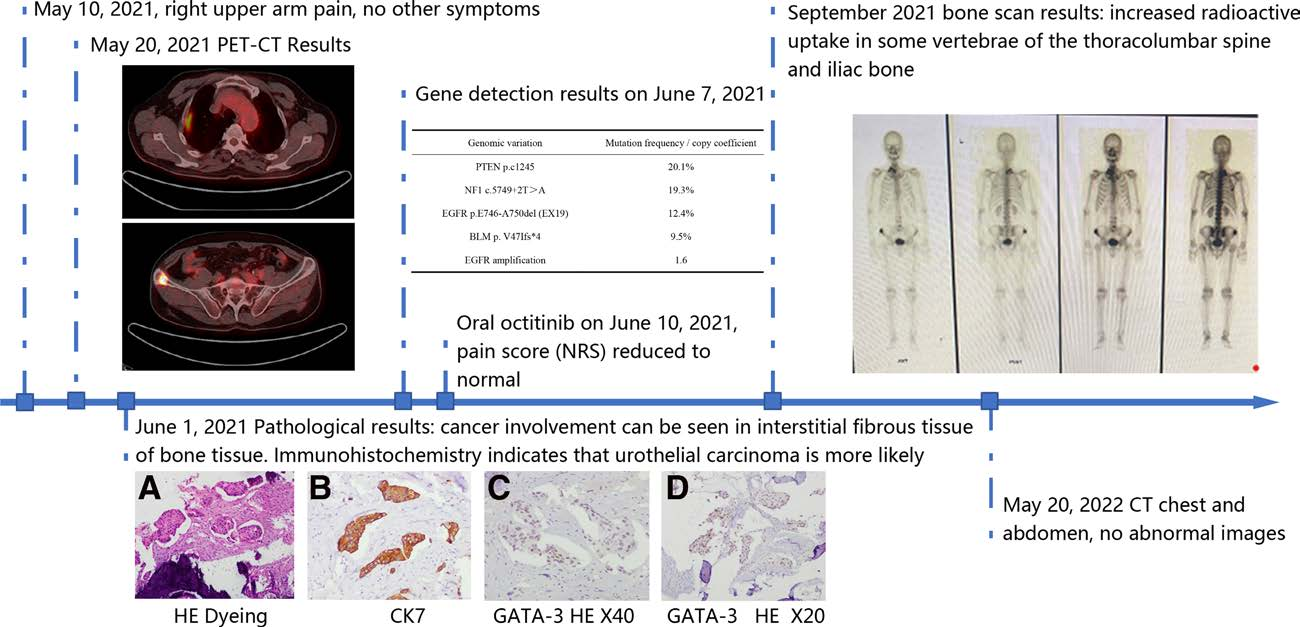
The timeline illustrates the diagnosis and treatment process of this patient. PET/CT scan: PET-CT demonstrated high FDG uptake in the right 1st and 2nd ribs and right iliac bone; Pathology diagnosis. (A): Gross appearance of the tumor; (B): H&E staining of iliac puncture tissue; (C–E): On the immunohistochemical examinations the cells were stained positive for CK7, GATA-3; Somatic genomic variation was conducted on June 7, 2021, demonstrated in the table; Bone scan results suggested increased radioactive uptake in some vertebrae of the thoracolumbar spine and iliac bone. FDG = fludeoxyglucose, PET/CT = positron emission tomography/ computed tomography.

Physical examination: normal development, moderate nutrition, listlessness, heart, lungs, and abdomen (–), positive tenderness in the right scapula area, and limited scapular joint movement. After admission, complete the relevant examinations. Radionuclide bone imaging examinations incidentally revealed osteogenic enhancement foci in the right 1st and 2nd ribs, thoracic 11 vertebrae, and right iliac bone, so we need to combine with other examinations to exclude the possibility of bone metastasis. The chest radiograph was normal. The results of 9 tumor markers for males showed: carcinoembryonic antigen 6.27 ng/mL, carbohydrate antigen 199 31.50 U/mL, and the remaining items are normal. Positron emission tomography/ computed tomography examination was performed to evaluate the range of metastasis. It demonstrated: The first and second ribs on the right, the thoracic 11 vertebral body and the right iliac bone were destroyed, and the fludeoxyglucose (FDG) metabolism was abnormally increased. The more obvious 1 was located on the second rib on the right whose standardized uptake value max was 11.72 and standardized uptake value avg was 4.91, it is recommended to exclude metastatic tumors from external examination. Old lesions in the upper lobe of the right lung. There is no abnormality in brain FDG-PET imaging and head CT scan. FDG-PET imaging of the neck and plain CT scan of the head showed no abnormalities. Abdominal FDG-PET imaging and head CT plain scan showed no abnormal changes. Degeneration of cervical, thoracic, and lumbar spine. May 2021, 17 CT-guided right iliac bone space puncture biopsy, June 2021, 01 pathological biopsy prompts: [right iliac bone space puncture tissue] There is cancer involvement in the interstitial fibrous tissue of the bone tissue, immunohistochemistry It is suggested that urothelial cancer is highly likely, and it is recommended to complete the clinical examination to clarify the primary tumor. carcinoembryonic antigen (–), CK (+), CK20 (–), CK5/6 (+), CK7 (+), GATA-3 (+), Ki67 (40%+), NKX3.1 (–), NapsinA (–), P40 (–), P63 (–), TTF-1 (–), Villin (–). Cystoscopy had no abnormalities. June 2021, 07 Gene test: [Iliac puncture tissue] (Fig. 1). Microscopic findings: tumor cells are arranged in nests, interstitial cells are seen to promote fibrous connective tissue proliferation and mucinous degeneration, tumor cells have large nuclei and deep staining, and the nuclear to pulp ratio increases. programmed cell death 1 ligand 1 immunohistochemistry (antibody type 22C3, detection method IHC): Tumor proportion score < 1%, combined positive score < 1.

Based on next-generation sequencing technology, 4 types of mutations (including point mutations, small indels, copy number variations, and currently known fusion genes) of 1021 genes related to tumor occurrence and development were detected (Table 1). The somatic genome variation is shown in Figure 1. History: He had no previous history of relevant tumors. He suffered from tuberculosis more than 20 years ago which was cured with by regular anti-tuberculosis treatment. He denied a family history of tumors and genetic disease. In summary, the diagnosis is clear: secondary malignant tumors of bone with unknown primary focus stage IV (EGFR19del, EGFR amplification), tumor mutation burden-L.

**Table 1:** Gene variation.

Detection range/genomic variation	Detection result and significance
1.Somatic variation: All exon regions of 312 genes, intron, promoter or fusion breakpoint regions of 38 genes, and some exon regions of 709 genes;	13 variants were detected, of which 5 were related to targeted drugs
2.Germline variation: All exons of 39 genes	0 variants were detected, of which 0 were related to targeted drugs
3.Genomic index: Tumor mutation load (TMB) and microsatellite instability (MSI)	Tumor mutation load - low TMB-L, 7.68 Muts/Mb, 70%. Microsatellite stability MSS

TMB = tumor mutation burden.

Treatment process: On June 10, 2021, oral Osimertinib 80 mg once a day was started, and bisphosphonates anti-bone destruction treatment was given on schedule. The patient insisted on taking the medicine with good compliance. After treatment, the patient’s pain improved significantly, with the numeric rating scale score reduced to normal from the previous 6 to 7. After 3 months, the bone scan was reexamined at the external hospital, which showed that multiple radiological concentrations in the right 1st and 2nd ribs, the right upper humerus, the *T*11 to *T*12 vertebrae, the *L*3 vertebrae, the *L*5 vertebrae, and the right iliac bone. After reviewing the whole-body CT scan in May 2022, no other primary tumor lesions were found, and the bone scan was rejected. Since the targeted treatment started 11 months ago, there has been no bone pain and other discomfort symptoms. Toxic and side effects: Rash grade 1, diarrhea grade 1, and recovered after symptomatic treatment. Treatment-related adverse events were graded according to the National Cancer Institute Common Terminology Criteria for Adverse Events, version 4.0 (https://ctep.cancer.gov/protocolDevelopment/electronic_applications/ctc.htm#ctc_archive). The treatment plan was satisfactory, and patients had a good quality of life.

## 4. Discussion

### 4.1. Overview of BMCUP

BMCUP is a type of heterogeneous tumor whose primary tumor cannot be identified by history collection, physical examination, and related laboratory examinations. Due to the unknown origin of the tissue and no specific targeted treatment measures, patients with BMCUP usually have a poor prognosis, with an average survival time of only 3 to 12 months.^[2–4]^ Adenocarcinoma is the most common pathological type of BMCUP, with poorly and moderately differentiated adenocarcinoma accounting for 64%, undifferentiated carcinoma accounting for 20%, neuroendocrine carcinoma accounting for 9%, and squamous cell carcinoma accounting for 7%.^[1]^ The most common site of BMCUP is the spine, followed by the pelvis and long bones.^[5]^ For the treatment of BMCUP patients, there is no clear and unified clinical treatment guideline, and chemoradiotherapy and surgery usually only play the role of palliative care. With the rapid development of genomics and molecular biology, finding out the corresponding therapeutic targets of tumors through molecular diagnosis and gene sequencing may provide a new treatment mode, to formulate individualized treatment plans.

### 4.2. EGFR gene mutation

EGFR is the expression product of the proto-oncogene HER-1 and has tyrosine kinase activity. EGFR mainly enters the nucleus through signal transduction. After entering, the transcription level of genes in the nucleus increases. Its overexpression leads to abnormal downstream signal transmission process, which accelerates cell proliferation and transformation, and can also resist apoptosis, prolong cell survival, and participate in tumors. The biological behaviors of cells, such as proliferation, invasion, and metastasis, are related to the occurrence, development, prognosis, and treatment response of tumors. A genetic mutation is a change in a single base pair in DNA or a gene. Gene amplification refers to the increase in copy number of some specific subchromosomal segments but does not include the amplification of the entire genome, chromosomes, arms, or large chromosomal regions.

EGFR gene mutation can occur in many epithelial tumors, among which the incidence in lung cancer is significantly higher than that in other tumor types, reaching 30.6%, followed by brain tumors and esophageal cancer. Studies have shown that the frequently mutated region of the EGFR gene in exons 18 to 21. The most common activating mutations are deletion mutations E746 to A750 in exon 19, L858R in exon 21, and exon 20 in exon 20. T790M drug resistance mutation.^[6–8]^ WJTOG3405 clinical study confirmed the predictive effect of EGFR mutation on the efficacy of tyrosine kinase inhibitor (TKI) (Tyrosine Kinase Inhibitors, tyrosine kinase inhibitor) drugs in the treatment of non-small cell lung cancer non-small cell lung cancer (NSCLC).^[9]^ Detection of the mutation status of related genes has become a predictor of the clinical efficacy of targeted drugs in non-small cell lung cancer and other tumor types.^[10]^

Abedi-Ardekani et al examined tumor tissue specimens from 152 patients with esophageal squamous carcinoma by direct sequencing which detected 1 case of EGFR19 exon E746 to A750 deletion with a mutation rate of 0.7%.^[11]^ Liu et al used the ARMS method to detect EGFR mutations in the specimens of 50 patients with esophageal squamous cell carcinoma.^[12]^ The results showed that there were 7 cases (14%) of EGFR mutation patients, of which 2 cases had exon 19 E746 to A750 deletion, and 5 cases were extra 21 Exon L858R point mutation. Ferry and other scholars also tested EGFR gene mutations in esophageal adenocarcinoma. The results demonstrated that 1 patient had a deletion of E746 to A750 in exon 19, and 1 had L858R point in exon 21 mutations, but it has not been reported whether patients with EGFR mutations are effective in treatment.^[13]^ A phase II TKI drug clinical study was conducted by Schilder et al detecting the mutations of exons 18 to 21 in 57 patients with ovarian cancer and primary peritoneal cancer.^[14]^ Two cases of EGFR mutations were detected (2/57, 3.5%), all were deletions of exon 19 (delE746–A750). One case was treated with TKI drugs (Gefitinib) to achieve complete remission, and the progression-free survival was 17.0 months.

### 4.3. EGFR gene amplification

The research found that EGFR gene amplification may also be a predictor of the clinical efficacy of EGFR-TKI.^[15]^ Another study showed that patients with EGFR gene mutation and amplification at the same time had better efficacy by using TKI. Therefore, the combined detection of gene amplification and gene mutation may be more helpful to the judgment of drug sensitivity and prognosis.^[16]^ However, the sequence of EGFR gene amplification and mutation is still unclear. Some researchers have proposed that gene mutation is a form of gene expression in the early stage of tumor and before invasion; while copy number increase, that is, gene amplification, is an end-stage, invasive form of manifestation. Relevant reports indicated that NSCLC patients with EGFR gene amplification have a higher probability of EGFR gene mutation, and EGFR gene mutation can cause an increase in the copy number of part of the gene.^[17,18]^ Cappuzzo and Eberhard also found that patients with EGFR gene amplification and higher copy number of non-small cell lung cancer have a better curative effect on EGFR-TKI, but they did not investigate the relationship between the EGFR gene amplification and targeted therapy in detail.^[19,20]^

## 5. Conclusion

Since the primary tumor of BMCUP cannot be identified, many studies have shown that individualized targeted therapy should target the genome of tumor rather than the tissue origin of tumor. Therefore, it is urgent to study the gene sequence of BMCUP. If multi-gene sequencing can be widely used, it will be beneficial to the discovery of molecular targets and the development of new drugs for specific mutations. It is possible that the future treatment mode will be directly based on the abnormality of the tumor cell genome, regardless of whether the primary tumor is well defined.

EGFR-based genetic testing has become a key measure for targeted therapy in NSCLC, and clinical benefits have been achieved by some mutant populations. However, similar tests for other tumor types and BMCUP have been relatively poorly studied, and the therapeutic efficacy is also unsatisfactory.

The current multi-gene detection technology is conducive to the discovery of relevant molecular targets. We hope that through this case report, we can have another therapeutic method for BMCUP. If relevant gene mutations are detected, corresponding targeted drugs can be selected to kill tumor cells and control the disease according to the “basket theory” to realize the “simultaneous treatment of different diseases” of advanced tumors.

However, more research needs to be devoted to the application of EGFR gene mutation or amplification in the treatment guidance and efficacy monitoring of other tumor types in the future which may even change the future treatment mode. The treatment will be based on abnormality in the genome of the tumor cell, regardless of whether its primary tumor is clear. To expand the beneficiary population of TKI targeted therapy, improve the quality of life of tumor patients to the greatest extent, and prolong the survival period.

## Acknowledgments

All authors disclosed no relevant relationships in conflict of interest.

## Author contributions

**Conceptualization:** Yu Zhou.

**Data curation:** Yu Zhou.

**Investigation:** Hong-Juan Du, Yu Liu, Yu Zhou.

**Methodology:** Hong-Juan Du, Yu Liu, Yu Zhou.

**Project administration:** Yu Liu.

**Software:** Hong-Juan Du.

**Supervision:** Yu Zhou.

**Validation:** Hong-Juan Du, Fang-Fang Chen, Yu Zhou.

**Visualization:** Hong-Juan Du, Fang-Fang Chen, Yu Liu.

**Writing – original draft:** Hong-Juan Du, Fang-Fang Chen.

**Writing – review & editing:** Yu Liu.
